# *TaylorActive –* Examining the effectiveness of web-based personally-tailored videos to increase physical activity: a randomised controlled trial protocol

**DOI:** 10.1186/s12889-015-2363-4

**Published:** 2015-10-05

**Authors:** C. Vandelanotte, C. Short, R. C. Plotnikoff, C. Hooker, D. Canoy, A. Rebar, S. Alley, S. Schoeppe, W. K. Mummery, M. J. Duncan

**Affiliations:** Physical Activity Research Group, School of Human Health and Social Sciences, Central Queensland University, Rockhampton, Queensland Australia; Freemasons Foundation Centre for Men’s Health, South Australian Health and Medical Research Institute, Faculty of Health Sciences, The University of Adelaide, Adelaide, Australia; Priority Research Centre for Physical Activity and Nutrition, Faculty of Health and Medicine, School of Medicine & Public Health, The University of Newcastle, Callaghan, Australia; Faculty of Physical Education and Recreation, University of Alberta, Edmonton, Alberta Canada

**Keywords:** Physical activity, Web-based, Online, Internet, Computer-tailoring, Text-based, Video-based, Intervention, Randomised controlled trial, Behaviour change

## Abstract

**Background:**

Physical inactivity levels are unacceptably high and effective interventions that can increase physical activity in large populations at low cost are urgently needed. Web-based interventions that use computer-tailoring have shown to be effective, though people tend to ‘skim’ and ‘scan’ text on the Internet rather than thoroughly read it. The use of online videos is, however, popular and engaging. Therefore, the aim of this 3-group randomised controlled trial is to examine whether a web-based physical activity intervention that provides personally-tailored videos is more effective when compared with traditional personally-tailored text-based intervention and a control group.

**Methods/design:**

In total 510 Australians will be recruited through social media advertisements, e-mail and third party databases. Participants will be randomised to one of three groups: text-tailored, video-tailored, or control. All groups will gain access to the same web-based platform and a library containing brief physical activity articles. The text-tailored group will additionally have access to 8 sessions of personalised physical activity advice that is instantaneously generated based on responses to brief online surveys. The theory-based advice will be provided over a period of 3 months and address constructs such as self-efficacy, motivation, goal setting, intentions, social support, attitudes, barriers, outcome expectancies, relapse prevention and feedback on performance. Text-tailored participants will also be able to complete 7 action plans to help them plan what, when, where, who with, and how they will become more active. Participants in the video-tailored group will gain access to the same intervention content as those in the text-tailored group, however all sessions will be provided as personalised videos rather than text on a webpage. The control group will only gain access to the library with generic physical activity articles. The primary outcome is objectively measured physical activity. Secondary outcomes include website engagement and retention, quality of life, depression, anxiety, stress, sitting time, sleep and psychosocial correlates of physical activity. Outcomes will be measured at baseline, 3, and 9 months.

**Discussion:**

This study presents an ideal opportunity to study the effectiveness of an isolated feature within a web-based physical activity intervention and the knowledge generated from this study will help to increase intervention effectiveness.

**Trial registration:**

Australian New-Zealand Clinical Trial Registry: ACTRN12615000057583. Registered 22 January 2015.

CQUniversity Ethics Project Number: H14/07-163

## Background

To prevent chronic disease, 30 minutes a day of moderate intensity physical activity has substantial positive effects on health and wellbeing [1, 2]. Yet, only 46 % of Australians meet the National Physical Activity Guidelines [3, 4]. This level of inactivity is costing Australia an estimated $1.6 billion in health care costs every year [5]. To change the prevalence of inactivity and make a considerable impact on population health interventions are needed that can reach a large number of people, change behaviour effectively (in short- and long-term) and be affordable when implemented in large populations. While this is a significant challenge, web-based interventions have considerable potential to achieve good outcomes in this context [6]. Over 80 % of Australians have access to high-speed broadband connections, and nearly 80 % of those use the Internet everyday [7]. This large uptake has virtually erased the traditional socio-demographic differences in Internet access (age, gender, education), and while a ‘digital divide’ may persist for certain population subgroups it is rapidly declining [7]. Therefore, the Internet is one of the most powerful media for reaching and connecting with people.

To date, numerous web-based physical activity interventions have been evaluated, however they predominantly demonstrate short-term effectiveness in increasing physical activity [6, 8]. Many studies report rapid declines in website usage as the interventions progress, which limits their long-term effectiveness [9, 10]. Low levels of website interactivity are also suggested to explain the modest participant engagement and retention rates in these studies [11, 12]. Websites that provide ‘tailored’, or individually adapted feedback about physical activity are highly interactive and show good effectiveness [13–15]. Compared with generic information users find tailored information more interesting and engaging because it is personally relevant [13]. Computer-tailored interventions can provide large numbers of people with individualised behaviour change information at low cost [15]. Compared with generic messages, tailored messages are more likely to be read and remembered, saved, and discussed with others [14].

Website intervention content is commonly presented as text-based information; this is problematic as there is evidence that text-based information is not effectively transmitted via the Internet. People do not fully read text-based information on the Internet, but rather ‘scan’ and ‘skim’ the content [16]. Internet-based reading behaviour is characterised by more time spent on browsing and scanning, keyword spotting, non-linear reading and more selective reading, while less time is spent on in-depth and concentrated reading [16, 17]. An eye-tracking study concluded that people did not read websites as they would read a book or newspaper. Instead, participants quickly scanned websites in an F-shaped pattern, in which they primarily focussed on the top-left quadrant of the page, and this was consistent across many different types of websites [18].

With the rise of broadband Internet connections, web-based video has become an increasingly popular part of users’ online experiences and it will soon surpass television as the most popular vehicle for delivering video-based content [19]. For example, 71 % of adults use video-sharing websites such as *YouTube* and 28 % of adults accesses these sites daily [20]. Videos have been used in previous health behaviour change websites; however their contents have not been tailored to individual recipients, mainly because the videos were merely supporting other intervention components [21–23]. Pilot studies that informed the current study by Soetens et al. [24] and Alley et al. [25], have demonstrated initial efficacy for using tailored videos, with participants in the video group rating the acceptability of the advice significantly higher than those receiving text-based feedback; they also spent significantly more time on the website. However, the methodological limitations of these studies (e.g. subjective measures, short follow-up periods, under powered samples) require that these outcomes be confirmed in an adequately powered and methodologically sound randomised controlled trial.

To fully harness the potential of computer-tailored websites they will need to be implemented in a new way. Popular websites are not only highly interactive, but they also provide most of their content in a visually attractive and engaging format (images or video). Health promotion websites are competing for attention with more popular websites, and to be successful, these sites will need to be equally interactive and engaging. Therefore, the current study will examine the effectiveness of a web-based physical activity intervention that provides personally-tailored videos and compare it to traditional personally-tailored text-based information and a control group. Secondary study aims are to examine mediators (e.g. psycho-social correlates) and moderators (e.g. socio-demographics) of intervention effectiveness, as well as to examine between group differences in changes to quality of life and participant engagement and retention.

## Methods

### Participants

Eligible participants will be adults who have broadband Internet access, speak and read the English language, live anywhere in Australia, and fail to meet existing national physical activity guidelines of engaging in 150 minutes of moderate to vigorous physical activity over five or more days of the week [26]. The ‘Physical Activity Readiness Questionnaire’ (PARQ) will be used to assess whether potential participants can safely increase their physical activity levels [27]. If this is not the case, and without prior medical clearance, they will be excluded from the study, as will pregnant women, those with a BMI under 17.5 and people with a self-reported physical impairment preventing them from being or becoming more physically active. This study has been approved by the Central Queensland University Human Ethics Committee (H14/07-163), and complies with the Helsinki Declaration.

### Recruitment

Five hundred and ten participants will be recruited through social media advertisements (e.g., Facebook), traditional media (e.g., newspaper, radio), e-mail and third-party databases (e.g. www.trialfacts.com). All advertisements will direct interested individuals to a specific recruitment webpage. On this recruitment page more information about the study can be found along with the participant information sheet. If people are interested in participating in the study they will be asked to complete an online screening questionnaire to assess their eligibility. This page will be able to instantly assess whether people are eligible to participate and provide an explanation for those who are not eligible. If eligible, people will additionally be asked to complete an online form to record contact details and provide informed consent with regards to their participation in this study.

### Procedure

When contact details of eligible people are received through the website, a project officer will make contact either through e-mail or phone (according to indicated preference) to verify the information provided. Subsequently, people will receive an Actigraph activity monitor using registered mail, as well as instructions on how to use the device, an Actigraph monitor log, a participant information sheet, and a return post-bag. They will be asked to wear the activity monitor for 7 consecutive days (and nights if comfortable) from the day of receipt. A project officer will contact potential participants to ensure the activity monitor has arrived and that they are wearing the device as instructed. At this time, the project officer will also schedule a time to conduct the self-reported baseline measures. The self-reported baseline measures will be administered via Computer Assisted Telephone Interviewing (CATI) by trained interviewers who are blinded to group allocation from the Population Research Laboratory (PRL) at the Central Queensland University (CQU). The CATI assessment will take approximately 30 minutes to be completed. When the activity monitor with valid data and Actigraph log sheet are returned to the university and the CATI assessment has been completed, participants will be randomised into one of three groups using a randomly generated sequence through freely available software (www.randomization.com). The same measurement procedures will be repeated at 3 and 9 months. These study procedures ensure the collection of high quality data without face-to-face contact. This better represents the reality of using health promotion websites and allows that participants can live anywhere in Australia.

### Study Design

**Fig. 1:**
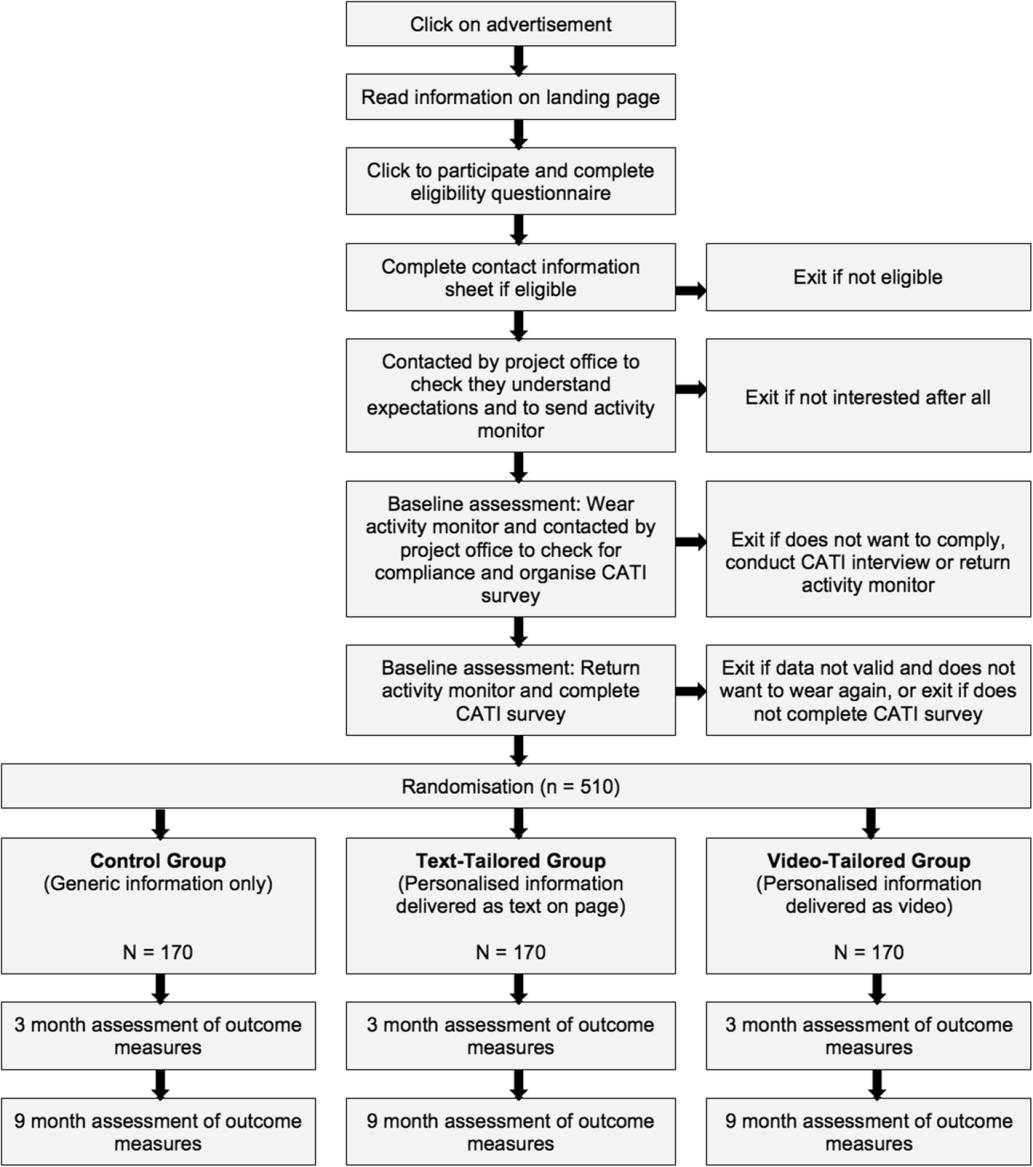
Study design

This is a three group randomised controlled trial with assessments at baseline, 3 months and 9 months (see Fig. 1). The same website will be used for all groups, but it will be programmed so that it only provides participants with access to the intervention features related to their allocated group. All groups will gain access to a static text-based library that contains generic physical activity information. The control group will only receive access to this library and will not have access to any other intervention features. Findings from previous studies indicate that static websites providing generic information are not effective in increasing physical activity, and can thus act as a control intervention [28]. In addition to having access to the generic library of articles, the ‘text-tailored’ group will have access to eight personally-tailored physical activity sessions that are delivered as personalised text on a webpage over a three-month period. The ‘video-tailored’ group will receive the same eight personally-tailored physical activity sessions, however they will be delivered as personalised videos. The videos will be released at the same schedule as the tailored text in the text-tailored group. All groups will receive regular reminders to return to the website at an identical schedule to control for the amount of contact between groups. This study is specifically designed to isolate the effect of video-tailoring and compare it with a text-tailored group and a control group. Using the same web-based platform for all groups ensures that website usage between groups is not confounded by non-specific website elements such as design, readability, usability and user-friendliness, as these may differ when using different websites and influence actual usage [29]. Having a web-based control group ensures that exposure to intervention contents across all groups can be objectively measured through website-usage statistics.

### Intervention

#### Intervention content

**Library:** The intervention library is populated with 19 brief articles (generally 2 A4 pages in length or shorter) about different aspects of physical activity and what to do to increase physical activity levels. Example topics include: ‘Are you physically fit?’, ‘Getting motivated’, ‘What is a healthy weight?’, ‘Making time to be active’, ‘Get started walking’, ‘Tips and tricks’, and ‘Why be active?’. Each article can be rated by users by way of a ‘5 star’ system and the most popular articles are displayed at the top of the list. The articles can also be downloaded as a PDF-file. This generic content is available for participants in all groups.

**Personally tailored feedback:** As the intervention content provided for the text-tailored and video-tailored groups is identical it will be described in one section for both groups. To generate the personalised content, participants are asked questions about how active they have been the previous week in conjunction with questions relating to individual, social, environmental and theory-based correlates of physical activity behaviour. Based on the answers of participants, and through applying IF-THEN algorithms, personally relevant physical activity content is automatically selected from a database. In this study, eight sessions with personalised feedback will be provided to participants, hence participants will need to complete eight brief surveys before receiving feedback. The website stores answers to previous sessions, and therefore, is able to provide feedback on progress towards meeting goals. The eight sessions are organised in a set order and the ‘next’ session can only be accessed when the ‘previous’ session has been completed. All sessions are released at a set time point and if participants do not access newly available sessions, they will receive up to 3 reminder e-mails and a phone call from project staff. When a session is made available participants can complete it as many times as they like, however the session will be ‘locked’ when the next session becomes available. Personal feedback from past sessions will be accessible throughout the intervention but it cannot be changed. This locking of session is needed to produce progress feedback that is credible (e.g., the changing of original ‘baseline responses’ would make future progress feedback incorrect).

The behaviour change content addresses the main constructs of multiple behaviour change theories that have demonstrated their effectiveness in guiding health behaviour change, including the Theory of Planned Behaviour [30], the Self-Determination Theory [31], and Social Cognitive Theory [32]. The following constructs have been addressed in one or more of the sessions: self-efficacy (similar to perceived behavioural control or need for competence), intrinsic and extrinsic motivation, goal setting, action planning, intentions, social support, attitudes, peripheral and central cues, need for relatedness, locus of control, barriers, benefits, knowledge, outcome expectancies, risk perception, problem solving, decision taking, coping, relapse prevention, feedback on performance. Physical activity behaviour will be assessed in all sessions using a modified version of the Godin-Shephard Leisure-time exercise questionnaire [33] that also includes questions on resistance exercises. In the first session participants will be asked to select one of five motivations to be physically active: 1) to improve or maintain good health, 2) to increase fitness, 3) to increase strength, 4) to lose weight, 5) to feel better (improve mood and/or reduce stress). The feedback and physical activity goals will be tailored according to those motivations. In sessions four and six participants will have the option to choose a different motivation to be active if they wish. Table 1 provides an overview of the content of the eight sessions.

**Table 1:** Overview of the content in the tailored sessions

Session	Variables (number of items)	Feedback topics (number of permutations)	Determinants targeted
Session 1	- First and last name (2)	- Introduction and overview of the program (1)	Knowledge, goals, intrinsic motivation, autonomy, self-efficacy, outcome expectations, intentions
	- Gender, age, height, weight (4)	- Your physical activity guideline according to selected goal (7)	
	- Physical activity (10)	- Physical activity feedback (Are you meeting your guideline?):	
	- Setting an activity goal (1)	- Maintaining good health (8)	
	- Coping Self-efficacy (6)	- Increasing fitness (17)	
		- Increasing strength (15)	
		- Losing weight (21)	
		- Feeling better (26)	
		- Setting a goal for the next week according to self-efficacy level (9)	
		- Conclusion according to selected goal (5)	
Session 2	- Physical Activity (10)	- Welcome back (1)	Knowledge, self-efficacy, attitudes, perceived behavioural control, goals, intentions
	- Barriers to being active (3)	- Physical activity guidelines refresher (5)	
	- Goal setting (2)	- Physical activity progress feedback (27)	
	- Action planning (2)	- Barriers to being active (27)	
		- Setting S.M.A.R.T. goals (7)	
		- What is an Action Plan & example? (15)	
		- Conclusion (1)	
Session 3	- Physical Activity (10)	- Welcome back & Action Plan completed (3)	Goals, self-efficacy, outcome expectations, attitudes, intrinsic motivation, perceived behavioural control
	- Weight (1)	- Physical activity progress feedback (includes graphs):	
	- Action Plan Feedback (2)	- Overall physical activity level (9)	
	- Positive outcomes of activity (1)	- Number of physical activity sessions (4)	
	- Coping self-efficacy (6)	- Moderate and vigorous physical activity (16)	
		- Vigorous physical activity (6)	
		- Resistance exercises (17)	
		- Physical activity benefits:	
		- Positive changes already noticed (10)	
		- Benefits of being active according to demographics (16)	
		- Boosting your confidence (6)	
		- Conclusion (1)	
Session 4	- Physical Activity (10)	- Welcome back & Action Plan completed (5)	Goals, self-efficacy, intrinsic motivation, goals, intentions
	- Weight (1)	- Physical activity progress feedback (includes graphs):	
	- Action Plan Feedback (2)	- Overall physical activity level according to confidence and motivation (27)	
	- Confidence to meet goal (1)	- Number of physical activity sessions (4)	
	- Motivation to meet goal (1)	- Moderate and vigorous physical activity (16)	
	- Internal locus of control (2)	- Vigorous physical activity (6)	
	- External locus of control (2)	- Resistance exercises (17)	
	- Positive self-talk (3)	- Your physical activity guideline according to new goal (7)	
	- Negative self-talk (3)	- How your thinking influences your behaviour (6)	
	- Rewarding yourself (1)	- Rewards (2)	
	- Resetting your activity goal (1)	- Conclusion (1)	
Session 5	- Physical Activity (10)	- Welcome back (1)	Self-efficacy, intrinsic motivation, goals, intentions, knowledge, self-efficacy
	- Weight (1)	- Physical activity progress feedback:	
	- Action Plan Feedback (1)	- Overall physical activity (9)	
	- Changing habits (2)	- Number of physical activity sessions (4)	
	- Barriers to being active (1)	- Moderate and vigorous physical activity (16)	
		- Vigorous physical activity (6)	
		- Resistance exercises (17)	
		- Your physical activity journey from session 1 to 5 (includes graph) (12)	
		- Your weight evolution from session 1 to 5 (27)	
		- Forming new habits (4)	
		- Relapse prevention according to biggest barrier (10)	
		- Conclusion (1)	
Session 6	- Physical Activity (10)	- Welcome back (1)	Self-efficacy, knowledge, goals, intrinsic motivation, autonomy, social support, subjective norm
	- Weight (1)	- Physical activity progress feedback:	
	- Positive social support (3)	- Overall physical activity (9)	
	- Negative social support (3)	- Number of physical activity sessions (4)	
	- Influence on others (1)	- Moderate and vigorous physical activity (16)	
	- Resetting your activity goal (1)	- Vigorous physical activity (6)	
		- Resistance exercises (17)	
		- Optimizing your activity intensity (includes graphs) (12)	
		- Your physical activity guideline according to new goal (7)	
		- Social support (20)	
		- Social sabotage (20)	
		- Encouraging others to be active (2)	
		- Conclusion (2)	
Session 7	- Physical Activity (10)	- Welcome back (1)	Self-efficacy, perceived behavioural control, attitudes, autonomy
	- Work-related physical activity (3)	- Physical activity progress feedback (includes graph):	
	- Active transport (4)	- Overall physical activity (9)	
	- Household activity (2)	- Number of physical activity sessions (4)	
	- Urbanisation (1)	- Moderate and vigorous physical activity (16)	
		- Vigorous physical activity (6)	
		- Resistance exercises (17)	
		- Active lifestyle:	
		- Introduction (2)	
		- Active Travel (11)	
		- Active at work (11)	
		- Active at home (9)	
		- Active environments (9)	
		- Conclusion (2)	
Session 8	- Physical Activity (10)	- Welcome back (1)	Self-efficacy, Intrinsic motivation
	- Weight (1)	- Physical activity progress feedback (includes graph):	
		- Overall physical activity (9)	
		- Number of physical activity sessions (4)	
		- Moderate and vigorous physical activity (16)	
		- Vigorous physical activity (6)	
		- Resistance exercises (17)	
		- Comparing your moderate and vigorous physical activity at session 1 and 8 (4)	
		- Your physical activity journey from session 1 to 8 (includes graph) (5)	
		- Your resistance-training from session 1 to 8 (includes graph) (3)	
		- Your physical activity sessions from session 1 to 8 (includes graph (1)	
		- Your weight journey from session 1 to 8 (includes graph) (16)	
		- Success stories (5)	
		- Conclusion and checklist to stay on track (1)	

**Action Plans:** Participants in both intervention conditions will be strongly encouraged to set up action plans, and will have the option to create one immediately after sessions two trough eight (hence, seven in total). The action plans are separate to the physical activity sessions and can be skipped without disrupting participants’ progress through the program (unlike sessions that have to be completed in order to progress through the program). Action plans or ‘implementation intentions’ are a self-regulation strategy in the form of a setting up a detailed plan that can lead to better goal attainment and help in behaviour modification [34]. Practically, it means that participants will be asked very specific questions on how they will meet their activity goals: what activity they will do, where they will do it, when they will do it, how often they will do it, how long will each activity session be, and with whom they will do it. Participants will be able to provide these details for up to four different activities within each action plan. At the start of creating an action plan participants are also asked to set long-, medium- and short-term goals to reach their physical activity objectives. When participants have completed all the questions the website will provide a plan that can be printed on a single page. Participants are encouraged to display the plan in a prominent place (e.g. the fridge). The prompt to complete an Action Plan will occur immediately after completing a physical activity session, and it will ‘lock’ just prior to the next physical activity session becoming available. During this time, participants can complete the action plan as many times as they like.

**Frequently Asked Questions:** A frequently asked question section will be available for all groups, however the content is tailored to the information provided to participants. For example, only participants in the video-tailored group will have access to questions in relation to video playback. In general this section will provide participants with the details on how the website works, and how they can progress through the different modules and action plans.

**About:** This section explains who developed the TaylorActive intervention and why. This was included to increase the perceived credibility of the website and to satisfy user’s need for relatedness [35].

#### Intervention Delivery

**Fig. 2:**
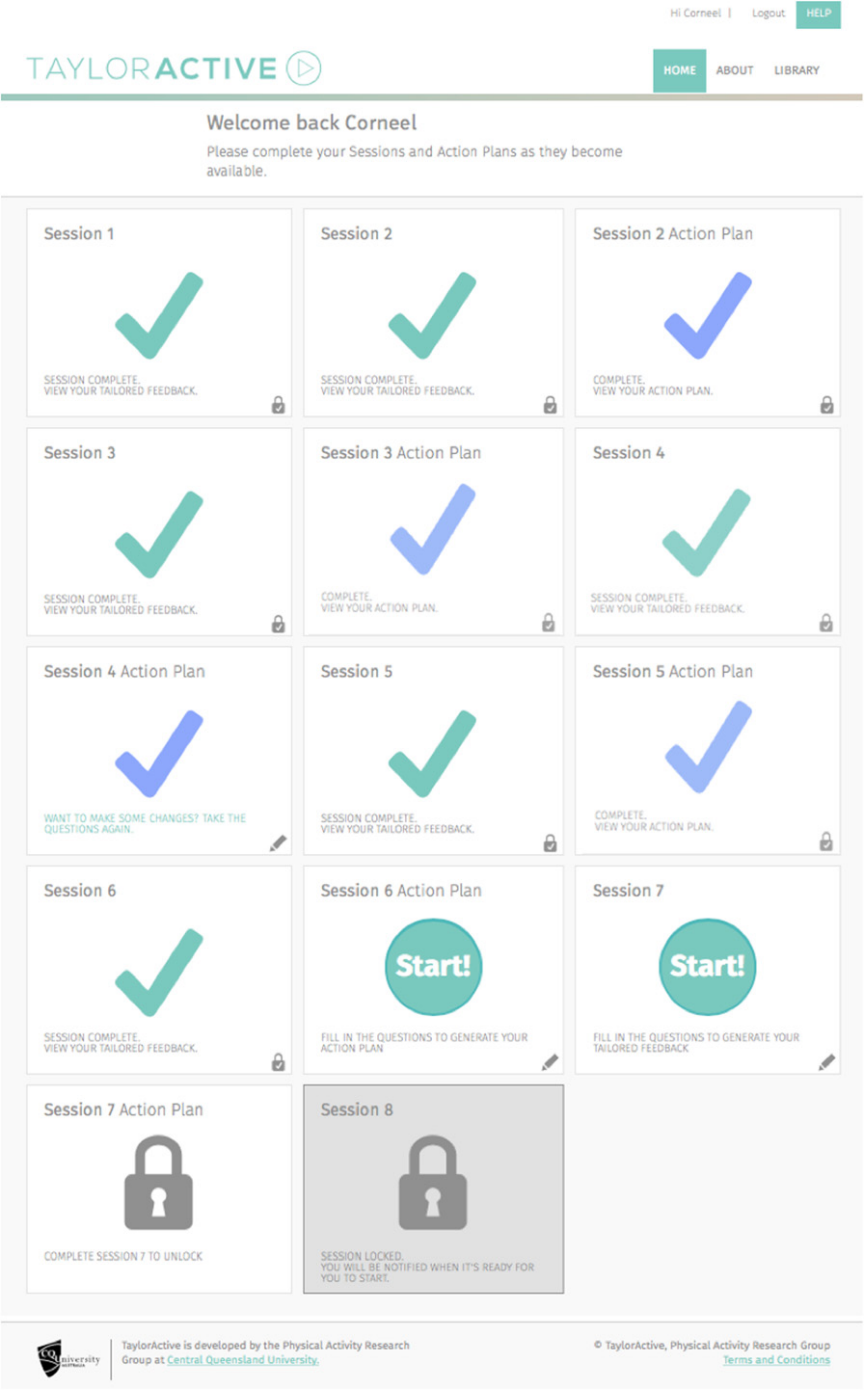
Screenshot of TaylorActive homepage.

**Fig. 3:**
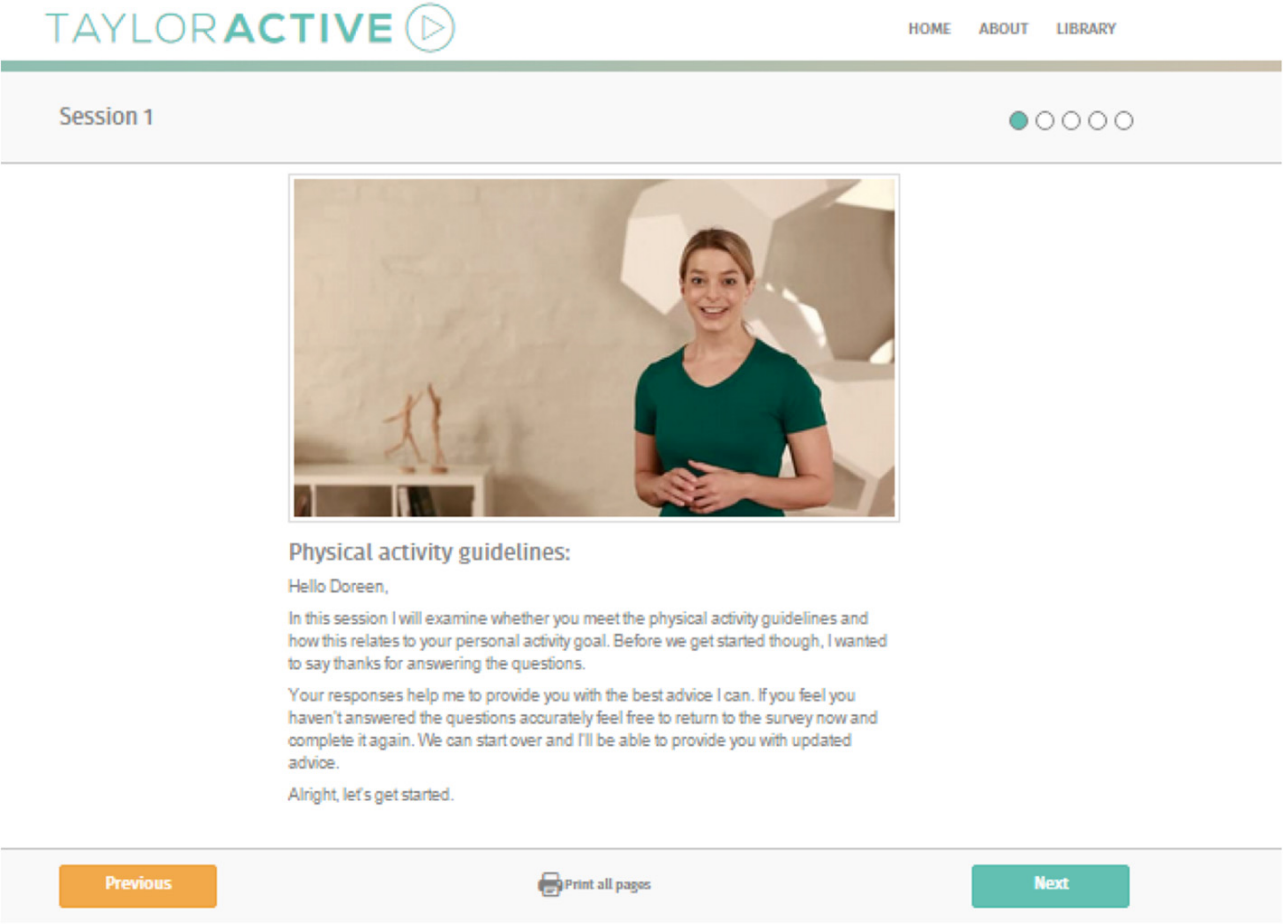
Screenshot of tailored text feedback with female presenter.

**Text:** The first four sessions will be delivered every 7 days (month one); the next four sessions will be delivered every 14 days (months two and three). As such, the total length of the program is 12 weeks (three months). If participants fall behind they will be able to ‘catch-up’ to get back onto the original timeline by accelerating the speed at which new sessions are ‘unlocked’. This design will encourage sufficient exposure to the intervention materials needed for behaviour change. Unlocking new website content over the course of the intervention provides participants with a good reason to return to the website on a regular basis and has been shown to increase participant engagement and retention [36]. The text-tailored feedback will be displayed as plain text on a webpage supplemented with graphs indicating progress where relevant. When participants first enter the website they can select one of two ‘Taylor’s’ (one male, one female) from a short description and photo who will guide them through the intervention and their journey to become more active. Throughout the different sessions photos of their selected Taylor will be shown to make the content look more engaging. Figs 2 and 3 show how this information will be delivered on the website.

**Fig. 4:**
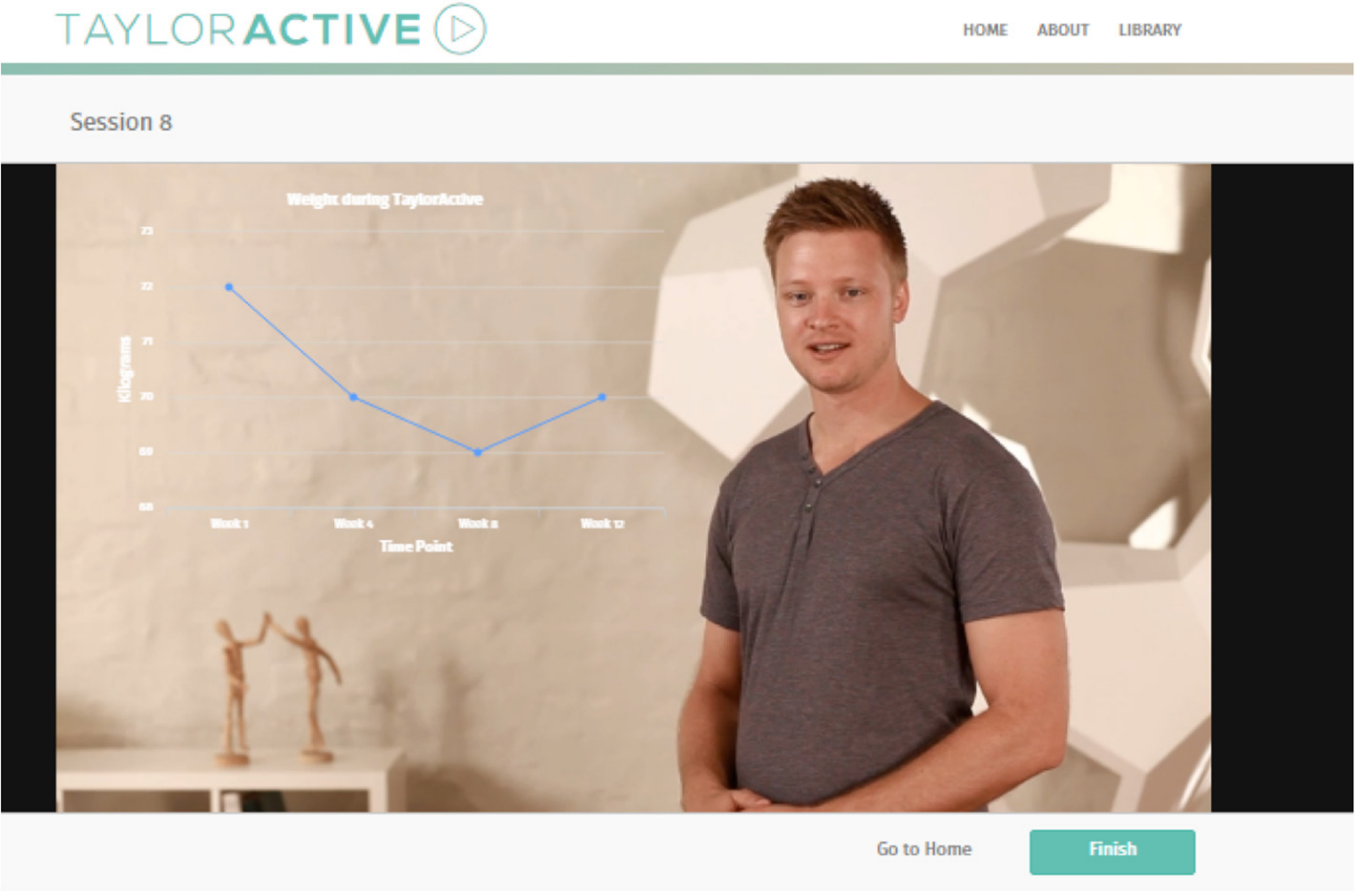
Screenshot of tailored video feedback with male presenter.

**Video:** The content in the video-tailored group will be ‘unlocked’ at the same schedule as the text-tailored group. The tailored content will be provided as a single seamless video for each session and will also be presented by one of two ‘Taylor’s’ (this time introduced by a short video upon first entering the website). The formative research conducted in preparation for this study indicated that the tailored videos should be relatively short [37] and therefore the amount of content provided at each session has been limited with this in mind. The video and other content was programmed so that it can be accessed using a wide range of popular web-browsers, as well as delivery platforms (i.e., computer, laptop, tabled, smartphones) and operating systems (i.e., Microsoft, Apple, Android). To avoid having to develop multiple systems, a website with a responsive design was implemented; the website reorganises itself to work well with screens of different sizes. Information that could easily be provided in the text-tailored feedback (e.g., participants name, BMI, minutes of vigorous physical activity), but that could not be pre-programmed into the videos will be provided as text layered on top of the video (i.e., an ‘overlay’) in an attempt to make the videos as personal as possible. Graphs showing progress over time will be provided in the same way. Figs 2 and 4 show how this information will be delivered on the website. To increase participant engagement each video ended with an element of fun ranging between 10 and 30 seconds (e.g., part of the decor coming to life and doing some physical activity).

### Measures

- **Physical activity** will be evaluated using both objective (ActiGraph activity monitor) and subjective (Active Australia Survey methods). The ActiGraph activity monitor (ActiGraph GT3X, http://www.theActiGraph.com) will be used to objectively measure weekly minutes of moderate and vigorous physical activity. The validity and reliability of the ActiGraph has been shown to be acceptable and comparable to other commercially available activity monitors [38]. Participants will be instructed on correct wear and fitting of the ActiGraph activity monitor. Participants will be asked to wear the unit over their right hip and firmly fastened with the supplied elastic waistband. Participants will also be asked to complete an activity monitor log detailing times the monitor was removed and activities undertaken when the Actigraph was not worn. Participants will be asked to wear the activity monitor on 7 consecutive days during waking and sleeping hours, except when swimming, bathing or participating in contact sports. Participants who are not comfortable sleeping with the device will be allowed to remove it during sleeping hours. Triaxial data will be collected in 1-second epochs along with step counts and inclinometry. When participants send back the ActiGraph the data will be inspected to ensure sufficient data has been collected. For the purposes of this study, valid wear time will be determined as at least 600 minutes wear time per day on 5 days [39]. Participants with invalid data will be asked to wear the activity monitor again. If, for the baseline measurement, participants refuse or return invalid data up to three times, they will be excluded from the study (see Table 2 for an overview of all measures). Finally, as mentioned above the Active Australia Survey will be used to subjectively measure physical activity. This survey provides contextual physical activity information as it assesses frequency and duration of walking for transport, walking for recreation, moderate intensity physical activity and vigorous intensity physical activity [40]. The Active Australia Survey has acceptable test-retest reliability and validity in the Australian adult population, and has been documented as a useful evaluative tool for detecting intervention related change in physical activity [41, 42].

**Table 2:** Overview of measurement tools

**Outcome**	**Measure**	**Number of items**	**Collection point (month)**
Physical activity	ActiGraph Accelerometer [38, 39]	8	0, 3, 9
	Active Australia Survey [40–42]	Objective	0, 3, 9
Sitting time	Workforce sitting questionnaire [43]	10	0, 3, 9
Sleep behaviour	Behavioral Risk Factor Surveillance		
	Screening Sleep Module [44]	4	0, 3, 9
	Pittsburgh Sleep Quality Index [45]	2	0, 3, 9
Depression, anxiety, stress	DASS 21 [46]	21	0, 3, 9
Quality of life	SF-12 [47]	12	0, 3, 9
Socio-cognitive correlates of physical activity	Individual items from several measures [48–53]	40	0, 3, 9
Physical activity environment	PANES [54]	12	0
Learning style	Newly developed items	4	0
Internet and technology use	Dimensions of Internet use [55]	1	0
	Items adapted from previous work [56]	7	0
Internet self-efficacy	Internet Self-efficacy scale [57]	8	0
Website statistics	Google Analytics	Objective	Continuous
	Additional newly developed items	4	3
User friendliness and usability	System Usability Survey [58]	16	3
	Additional newly developed items	5	3
Delivery mode usability and preference	Items adapted from previous work [24]	9 for text-group	3
		14 for video-group	
Advice acceptability	Items adapted from previous work [59]	16	3
Demographics	Commonly used items	13	0
- **Sitting Time** will be measured using the ‘Workforce Sitting Questionnaire’ [43]. All participants will report time (hours/minutes) spent sitting on usual working and non-working days in relation to work, transport, TV use, computer use and other leisure-time sitting. One question also measures the number of days participants usually work in a week. This survey has demonstrated adequate test-retest reliability and validity [43].
- **Sleep behavior** will be assessed using items from both the Behavioral Risk Factor Surveillance Screening Sleep Module [44] and the valid and reliable Pittsburgh Sleep Quality Index [45]. This study assesses average hours of sleep obtained, snoring behaviour, unintentionally falling asleep during the day, nodding off while driving, whether one feels rested and levels of sleep quality. Additionally, an objective measure of sleep will be collected in those participants that are willing to sleep with the Actigraph accelerometer on their waist (as this may be uncomfortable for some participants the study protocol allows removing the Actigraph during sleeping hours as it measures a secondary outcome only). Total sleep time, sleep efficiency, number of awakenings, sleep onset and sleep offset will be extracted from the Actigraph data using established protocols.
- **Depression, anxiety and stress:** The 21-item Depression, Anxiety and Stress Scale (DASS21) will be used to assess mental health outcomes. The DASS21 provides a quantitative measure of distress symptom severity; it is not a measure of clinical diagnosis. Three 7-item subscales measure depression, anxiety and stress. These scales have demonstrated acceptable validity and reliability in community samples of adults [46].
- **Quality of life** will be measured through the Short-Form Health Survey (SF-12) which includes questions on physical and mental health. The SF-12 is widely used, valid and reliable [47].
- **Socio-cognitive correlates of physical activity:** In line with the theoretical underpinnings of the intervention the following constructs will be measured using previously used and reliable measures regarding aerobic physical activity: attitudes (3 items measuring instrumental aspects; 3 items measuring affective aspects [48]); outcome expectancies (9 items [49]); subjective norm (2 items measuring injunctive component, 1 item measuring descriptive component [48, 50]); perceived behavioural control (2 items [48]); intentions (5 items [48]); barriers self-efficacy (5 items [51]); and action planning (4 items [50, 52]). The following correlates will also be measured specifically for resistance-based activity (using selected items from the measures above, adapted to resistance-training behaviour instead of aerobic physical activity): attitudes (2 items), subjective norm (1 item), perceived behavioural control (2-items), intentions (1-item), and action-planning (2-items). In addition, habit (4 items from the Self-report Habit Index [53]) and physical activity motivation (this item refers to the content of the intervention where participants can choose to be active for health, weight loss, strength, fitness or feeling better) will be assessed.
- **Perceived neighborhood environment:** To assess the influence of participant’s neighborhood environment on physical activity 12-items from the Physical Activity Neighborhood Environment Scale (PANES) will be used [54]. It assesses land mix use, street connectivity, residential density, pedestrian infrastructure, aesthetic qualities, safety from traffic and crime, and access to recreation facilities. The measure has shown to be valid and reliable [54].
- **Learning style:** For the purpose of this project we developed 4 items that assess whether participants prefer to process information and learn new things using text or video. We included these items as it may be that the study outcomes are influenced by how people prefer to acquire new information. The items are: When processing new information, do you prefer to a) watch and listen to online videos, b) read written information on a webpage, c) no preference; When learning about new things, do you prefer to a) watch and listen online videos, b) read written information on a webpage, c) no preference; I find it easier to understand a) spoken instructions, b) written instructions, c) no preference; When thinking about something do you usually think in a) words, b) pictures, c) both.
- **Internet and technology use:** Four items will be used to assess how frequently participants use the Internet, how much time they spend using the Internet, what purpose they use the Internet for (adapted from Blank and Groselj, [55]), and how fast their Internet connection is. A further 3 items will be used to assess technology use, e.g. tablets, smartphones, VOIP. Items will include: ‘What technologies do you use?’, ‘What technologies would you prefer to receive health behavior change information?’, ‘How often do you use social media websites?’. These items are based on previously published work [56].
- **Internet self-efficacy:** Participants’ confidence in their ability to execute tasks and trouble shoot problems with the Internet will be measured using the Internet Self-Efficacy Scale. This measure includes 8 items to assess a user’s understanding of Internet hardware and software, confidence in gathering information using the Internet and learning skills to use Internet programs, and ability to troubleshoot and resolve Internet problems. The scale has shown good reliability and internal consistency [57].
- **Website statistics:** Website usage and retention will be measured using the Google Analytics web traffic analysis platform, as well as by monitoring the generation of new user content. Features monitored will include (but are not limited to): number of logins, time on website, pages visited, sessions completed, action plans completed, and number library articles read. Though the least engaged participants will have completely stopped using the website (‘non-usage’ attrition), they might still complete the CATI assessments; as such, they would not have dropped from the intervention study (‘drop-out’ attrition) [9]. Both forms of attrition will be examined across groups. Website usage will be monitored for the entire study duration. Four additional survey items will also assess exposure to intervention contents, as well as whether intervention content was shared or discussed with others.
- **Website usability:** The overall usability of the website and intervention will be investigated using the System Usability Scale [58]. This scale has demonstrated acceptable reliability and validity for measuring usability of websites [58]. Five additional items will assess the usefulness of the website in terms of its ability: to increase confidence in becoming physically active; overcome barriers to be active; make plans to be active; increase support to be active; stay motivated to be active.
- **Delivery mode usability and preference:** To assess participant perceptions about how the tailored physical activity advice is delivered to them a number of delivery mode usability and acceptability items will be asked (8 for the text-group and 13 for the video-group). Example items are: ‘I found the graphs displayed in the text just right’, ‘I found the personalized text boring to read’, ‘I found the people presenting the information in the videos just right’, ‘The pace by which new information was presented in the videos was too slow’ (all items are assessed on 5-point agree-disagree Likert scales). Delivery-mode preference will assess how participants prefer the intervention is delivered to them (either video- or text-based), so that participants can be categorised as ‘matched’ or ‘mismatched’ with their preference after randomisation and its influence on intervention effectiveness examined. These items were previously used in a study by Soetens et al. [24].
- **Advice Acceptability:** A 16-item questionnaire will be used to assess whether the personalized contents of the physical activity feedback were perceived as acceptable and credible. This questionnaire is based on previously published work where advice acceptability of similar interventions was assessed [59]. Example items are: ‘The physical activity advice was personally relevant’, The physical activity advice met my expectations’ (all items are assessed on 5-point agree-disagree Likert scales).
- **Demographics:** The following demographic factors will be assessed: age, sex, weight, height, marital status, ethnicity, education, work status (e.g., retired), occupational category (e.g., blue collar), weekly work hours, income, postcode, and residential environment (e.g. rural area).

### Sample size

Sample size for the study is based on the primary outcome of change in moderate to vigorous intensity physical activity (min/week) measured by Actigraph accelerometry. Several reviews of web-based physical activity interventions suggest that most studies have small to moderate effects on change in physical activity and have high dropout rates; up to approximately 30 % [6, 8]. Hence, to detect a small to moderate difference in minutes of physical activity per week between groups (video-tailoring, text-tailoring and control), at the study’s primary time point of 3 months, 130 participants per group will be required to achieve 80 % power using an alpha level of 0.05. The number of participants per group has been inflated by 30 % (170 participants per group) to account for drop out. Hence, a total of 510 participants will be recruited into the RCT.

### Statistical Analyses

Generalized Linear Mixed Models (GLMM) will be used to analyse the data. GLMM analyses examine changes from baseline to each follow-up time point for each intervention group compared to the control group while accounting for within-person nesting of variables across time [60]. Data will be tested for linearity and normality to ensure the proper statistical techniques are applied. The most appropriate approach to handling missing data will be applied depending on the pattern and amount of the missing data. Linear regression and generalised linear models will be used to analyse moderating and mediating effects of intervention effectiveness, within the limitations of our sample size [61].

## Discussion

This study presents an ideal opportunity to study the effectiveness of a specific component within a web-based physical activity intervention. In recent years a growing number of randomized controlled trials have examined the effects of web-based health behavior change interventions [6, 8]. However, these interventions usually have a plethora of different intervention components making it impossible to attribute effectiveness to specific elements within the intervention [62]. Without more knowledge of what components do and do not work, it will be difficult to make progress and improve intervention effectiveness. Previous studies have emphasized the need for research examining the effectiveness of intervention components in isolation [63], and this is what our study will do. As such, we expect that the findings from this study will provide increased understanding of the benefits of new web-based technologies. It is anticipated that the personalized videos will be more engaging compared to personalized text-based information, which will lead to higher overall participant engagement and retention into the intervention. Higher participant engagement has shown to result in higher levels of behavior change [64] and ultimately this will lead to improved health outcomes. Strengths of our study are the use of objective measures to assess physical activity outcomes and web-usage outcomes, a large sample size with sufficient analytical power, a standardized exposure and contact across groups, no face-to-face contact with participants which better mimics real implementation of web-based interventions, and numerous measures to assess secondary outcomes, such as for example: ‘how do environmental attributes moderate intervention effectiveness?’.

## Abbreviations

AAQ: Active Australia survey
BMI: Body mass index
CATI: Computer assisted telephone interview
CQU: Central Queensland University
DASS21: Depression anxiety and stress scale 21
GLMM: Generalized linear mixed models
PANES: Physical activity neighbourhood environmental scale
PARQ: Physical activity readiness questionnaire
PRL: Population research laboratory
RCT: Randomised controlled trial
SF12: Short form 12 (Quality of life measure)
